# Tuberculosis Coalescent Mastoiditis: A Report of Two Rare Cases

**DOI:** 10.7759/cureus.77875

**Published:** 2025-01-23

**Authors:** Laura S Carneiro de Andrade, Luma T Nunes, Hardynn Wesley S Rocha Tavares, Vítor Yamashiro R Soares

**Affiliations:** 1 Department of Otolaryngology, Head and Neck Surgery, Hospital Getúlio Vargas/Faculty of Medicine of the State University of Piauí, Teresina, BRA

**Keywords:** canal wall down mastoidectomy, chronic otitis media, mycobacterium tuberculosis infection, tuberculosis mastoiditis, hearing loss

## Abstract

Tuberculous otomastoiditis is a rare disease. Diagnosis generally occurs late when the patient already has irreversible sequelae. Two cases are reported with an unusual presentation of coalescent mastoiditis caused by *Mycobacterium tuberculosis*. The patients underwent several antibiotic treatments without clinical improvement, evolving temporal bone erosion and severe/profound hearing loss. Maintaining a high degree of suspicion regarding this clinical entity is important to obtain an early diagnosis, contributing to better care for these patients and avoiding serious complications.

## Introduction

Tuberculous otomastoiditis is a rare form of infection of the middle ear and mastoid air cells by *Mycobacterium tuberculosis*. It corresponds to 4% of cases of tuberculosis in the head and neck region and only 0.05-0.9% of cases of chronic otitis media [1]. The clinical presentation resembles chronic non-tuberculous otitis media but without response to drug treatment [2]. As a result of low suspicion, the diagnosis of tuberculous mastoiditis is often delayed. Some signs and symptoms draw attention to tuberculous mastoiditis, such as painless otorrhea with multiple perforations of the tympanic membrane, granulation tissue in the middle ear and mastoid accompanied by bone erosions, peripheral facial paralysis, and early hearing loss incompatible with clinical findings [3]. Inadequate management causes complications and a high rate of morbidity for the patient [4,5].

The present study describes two cases with a rare presentation of coalescent tuberculous otomastoiditis with the main aim of spreading knowledge regarding this pathology to optimize the diagnosis and treatment of patients.

## Case presentation

Case 1

**Figure 1:**
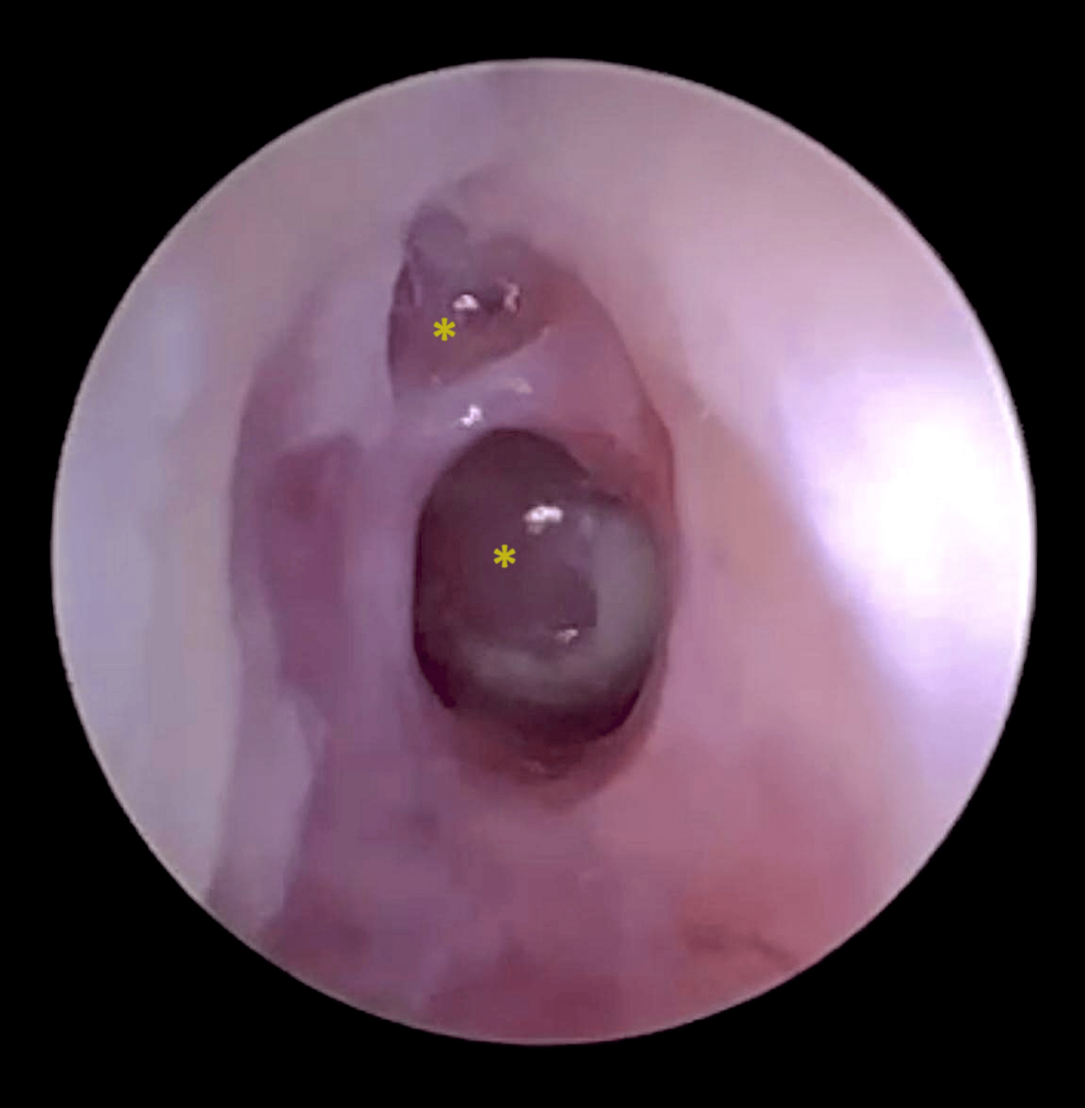
Otoendoscopy of the right ear showing a thick tympanic membrane with double perforation (asterisks) and hyperplasia of the middle ear mucosa.

A 28-year-old male patient was admitted to a tertiary hospital for investigation of persistent abdominal pain and discharge in the right ear. The otorrhea was purulent, painless, and refractory to multiple antibiotic regimens. The patient also had hypoacusis and continuous tinnitus for six months on the ipsilateral side. There were daily feverish episodes and unquantified weight loss. He reported smoking, alcohol consumption, and use of illicit drugs. Right otoscopy revealed double tympanic perforation and hyperplasia of the middle ear mucosa (Figure 1). Empirical antibiotic therapy (meropenem and vancomycin) was initiated until the etiological investigation was completed.

**Figure 2:**
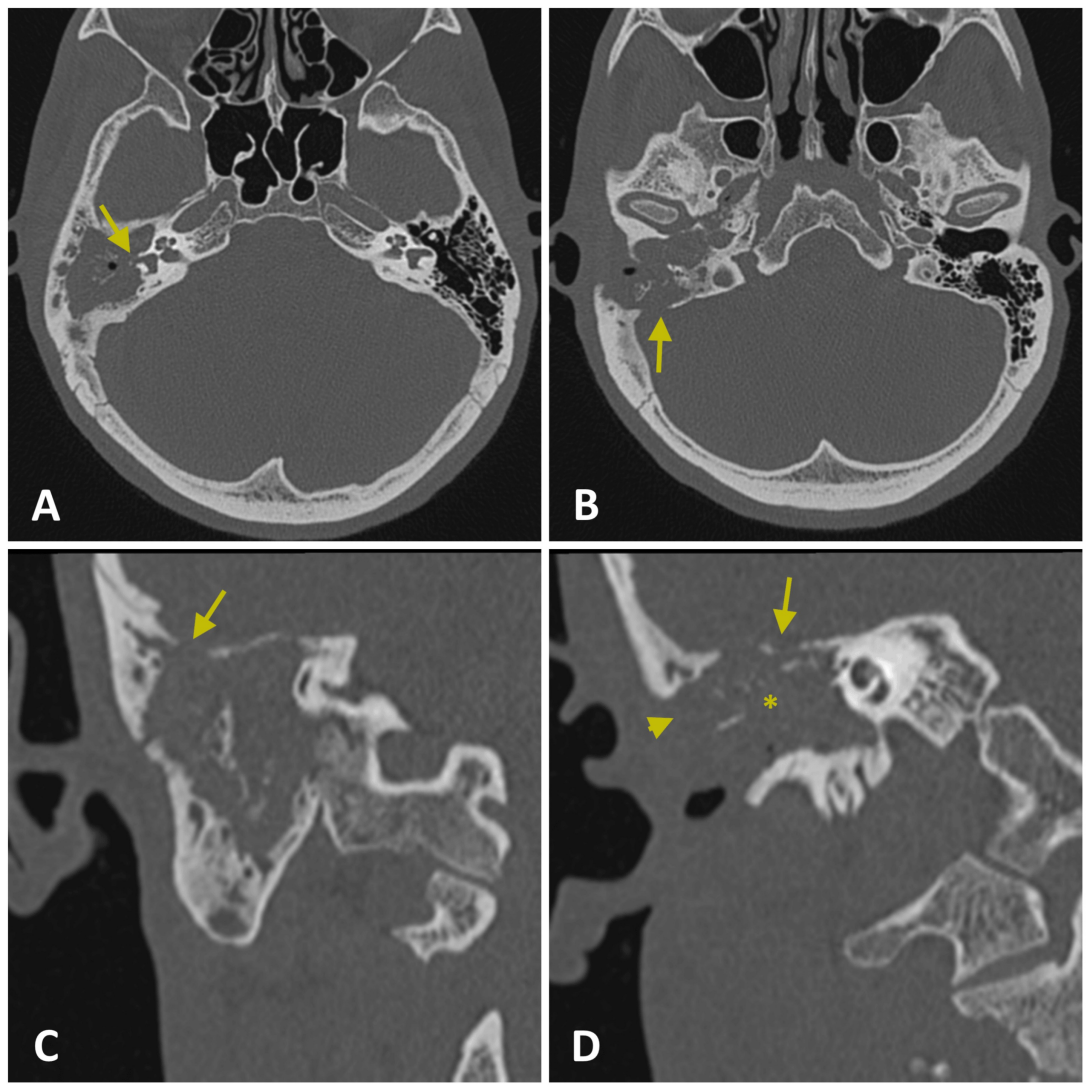
HRCT of the temporal bone detects coalescent mastoiditis with extensive bone erosion and bone sequestration on the right, mastoid cavity occupied by soft tissue density. Axial section showed (A) erosion of the lateral semicircular canal (arrow) and dehiscence of the tympanic portion of the nerve facial, (B) and with sigmoid sinus dehiscence (arrow). The coronal section showed (C) discontinuity of the middle fossa (arrow), (D) discontinuity of the tympanic tegmen (arrow), erosion of the external auditory canal (asterisk), and erosion of the cortical bone of the mastoid (arrowhead). HRCT: high-resolution computed tomography

**Figure 3:**
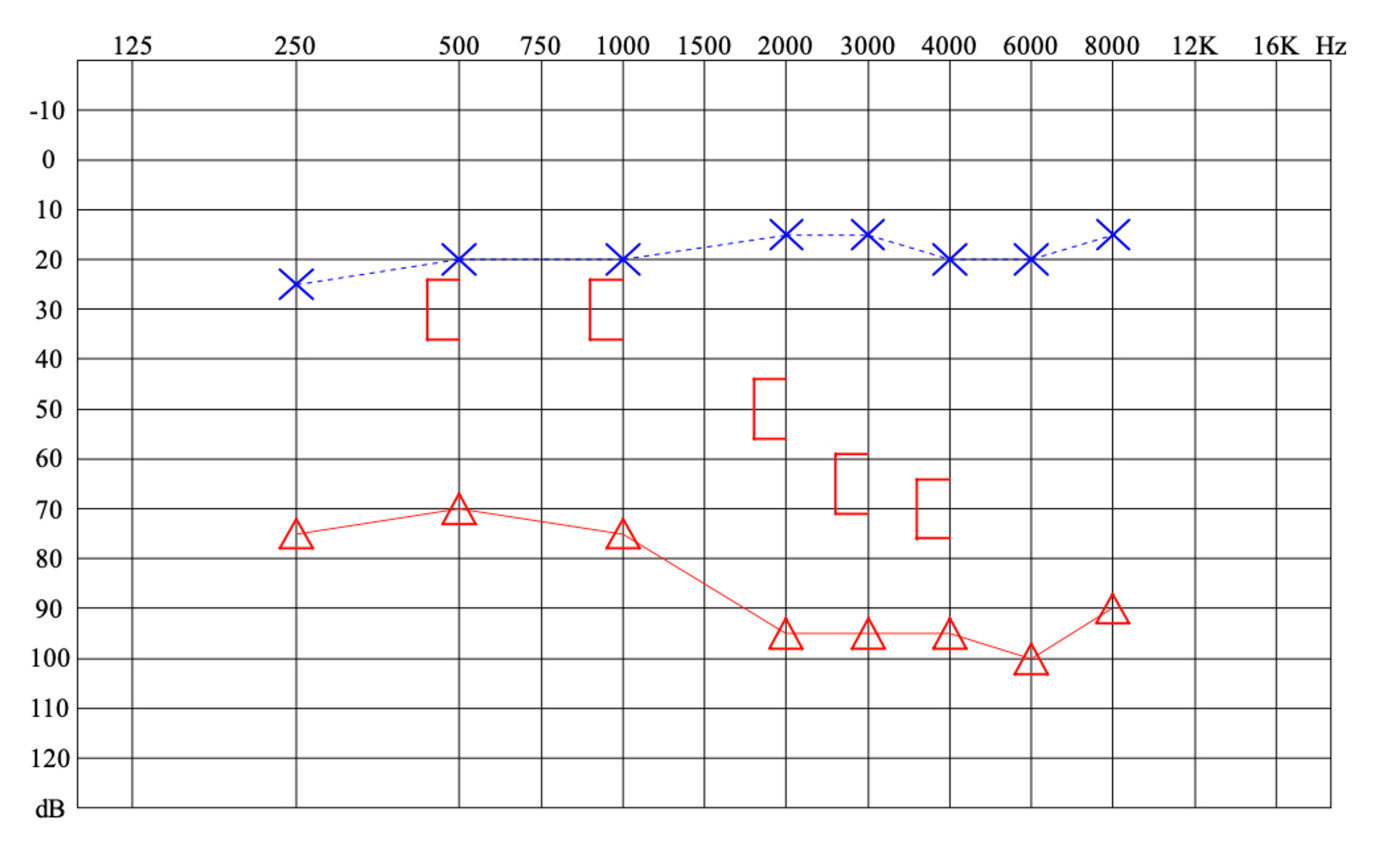
Pure tone audiometry demonstrates normal thresholds in the left ear (blue) and severe mixed hearing loss in the right (red).

High-resolution computed tomography (HRCT) of the mastoids showed the presence of soft tissue density in the mastoid, middle ear, and external auditory canal (EAC) on the right. There was bone erosion of the sigmoid sinus wall and petrous apex, in addition to dehiscence of the lateral and superior semicircular canals. The mastoid bone trabeculae had been destroyed with the formation of coalescent mastoiditis (Figures 2A-D). Contrast-enhanced magnetic resonance imaging of the temporal bone showed a hypersignal on T2 and an intermediate signal on T1 located in the mastoid region, tympanic cavity, and EAC, with peripheral enhancement by gadolinium contrast medium and bone destruction in these regions. Pure tone audiometry showed severe mixed hearing loss in the right ear and normal thresholds in the left ear (Figure 3).

Chest HRCT showed multiple solid nodules scattered throughout the lungs, with consolidations and cavitations. Sputum smear microscopy showed the absence of acid-fast bacilli (AFB). The rapid molecular test for tuberculosis (RMT-TB) detected DNA from *Mycobacterium tuberculosis*, which is sensitive to rifampicin. The diagnosis was paucibacillary pulmonary tuberculosis. The patient was treated with a therapeutic regimen for tuberculosis with rifampicin, isoniazid, pyrazinamide, and ethambutol.

**Figure 4:**
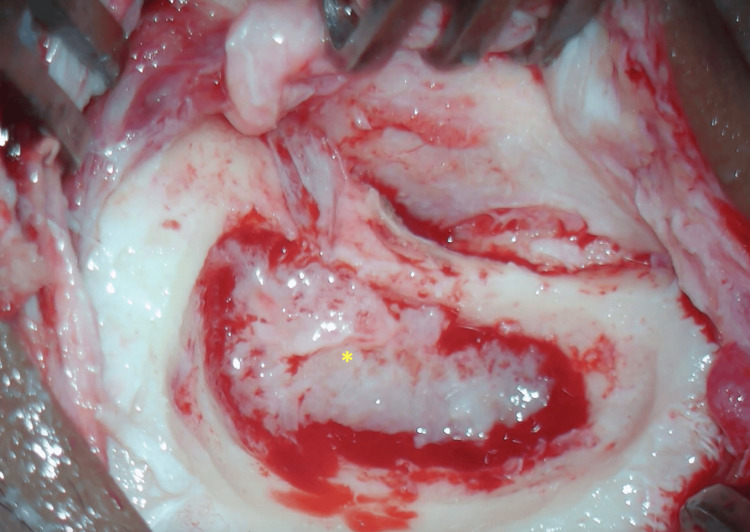
Intraoperative image with granulation tissue with a friable and pale appearance (asterisk) occupying the right mastoid.

After completion of isolation, the patient underwent canal wall-up tympanomastoidectomy on the right. There was erosion of the cortical bone and coalescence of the mastoid cells. The cavity was filled with inflammatory tissue that appeared pale and friable (Figure 4). There was a dehiscence of the sigmoid sinus and the dura mater of the posterior fossa, which appeared thickened. The lateral semicircular canal was eroded and covered by inflammatory tissue that was preserved due to the risk of iatrogenic labyrinthine fistula. The tympanic portion of the facial nerve was also dehiscent and surrounded by inflammatory tissue that also filled the entire middle ear. The ossicular chain was absent. This inflammatory tissue was used for histopathological evaluation, in addition to the AFB test, fungal, bacterial, and mycobacterial culture, and RMT-TB.

The histopathological examination showed nonspecific chronic osteomyelitis. The culture showed no growth of bacteria or fungi. Bacilloscopy was positive for AFB, and RMT-TB detected DNA from Mycobacterium tuberculosis, which is sensitive to rifampicin. The patient was also diagnosed with coalescent tuberculous otomastoiditis concomitant with pulmonary tuberculosis.

The patient was treated with a therapeutic regimen for tuberculosis with rifampicin, isoniazid, pyrazinamide, and ethambutol, as recommended by the Manual of Recommendations and Control of Tuberculosis in Brazil [6]. In the first two months, the patient received rifampicin 450 mg/day, isoniazid 225 mg/day, pyrazinamide 1200 mg/day, and ethambutol 825 mg/day. Subsequently, the patient received rifampicin 450 mg/day and isoniazid 225 mg/day for four months in the maintenance phase. After two years of follow-up, the EAC was patent, the tympanic membrane was intact, and there were no clinical signs of recurrence.

Case 2

A 25-year-old male patient is complaining of progressive hearing loss, dizziness, tinnitus, otalgia, and otorrhea in the right ear with no response to topical antibiotics. The symptoms started four months prior. Facial expression was preserved. He also had mild dyspnea, fever, and weight loss (22 lb). Otoscopy showed purulent secretion, edema, and the presence of a polyp filling the right EAC.

**Figure 5:**
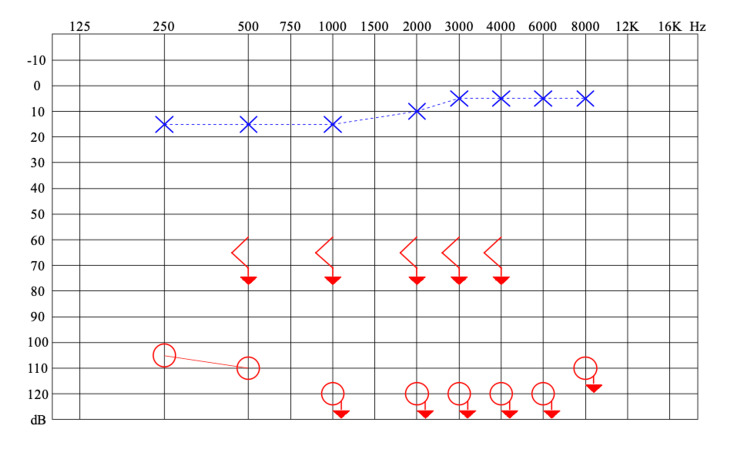
Pure tone audiometry showing normal thresholds in the left ear (blue) and profound sensorineural hearing loss in the right (red).

**Figure 6:**
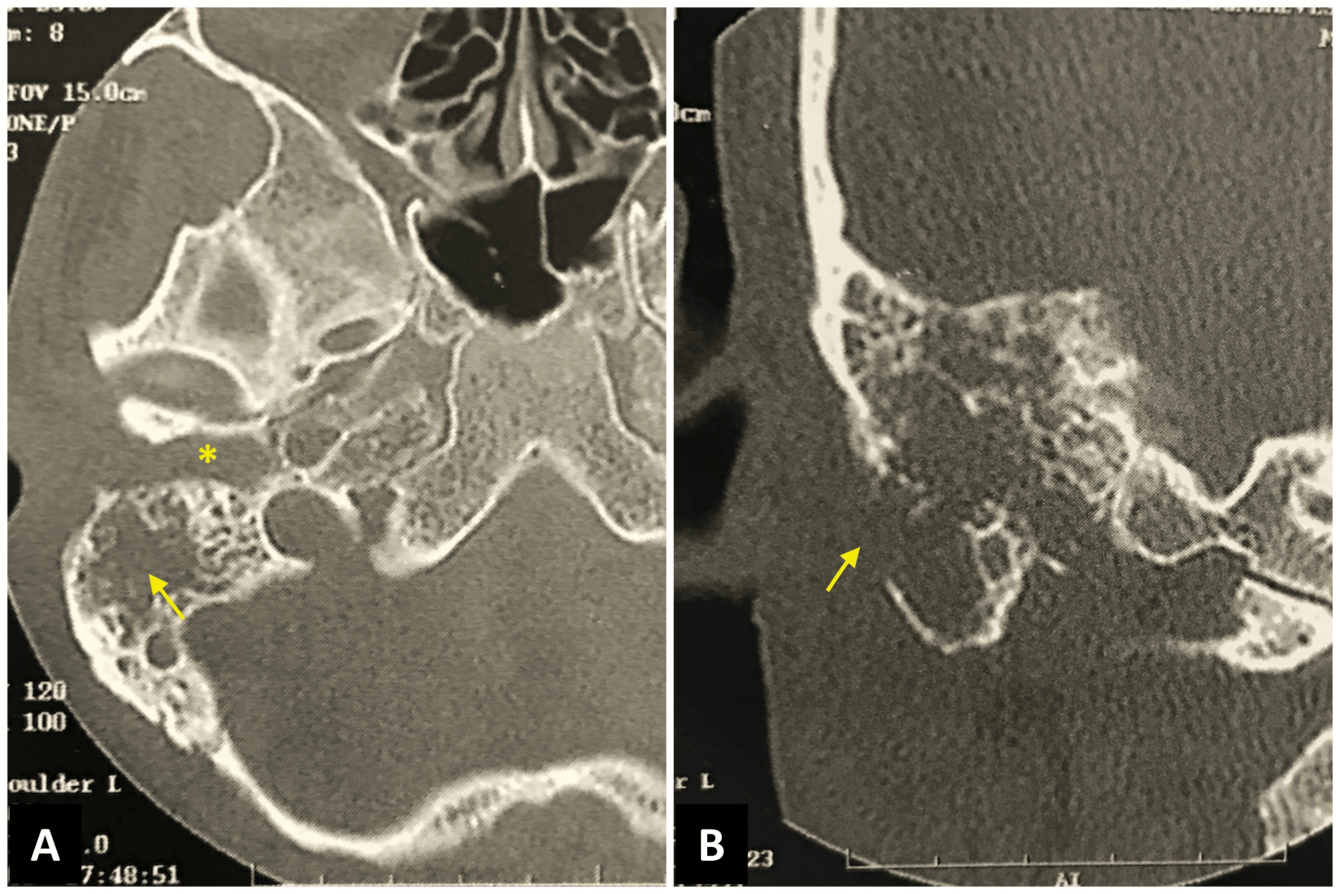
HRCT of the temporal bone detects coalescent mastoiditis on the right. (A) Axial section with erosion and bone sequestration of the mastoid trabeculae (arrow) and soft tissue density occupying the mastoid cavity, middle ear, and external auditory canal (asterisk); (B) coronal section showing discontinuity of the mastoid cortical layer (arrow). HRCT: high-resolution computed tomography

Audiometry tests revealed profound sensorineural hearing loss in the right ear and normal hearing in the left ear (Figure 5). The mastoid HRCT showed soft tissue density occupying the entire EAC, the middle ear, and the mastoid on the right. There was discontinuity of the cortical layer and bone trabeculae of the mastoid with the formation of coalescent mastoiditis (Figures 6A, 6B). The cervical ultrasound examination showed an enlarged retroauricular lymph node. The HIV serology was negative. The chest X-ray examination identified pulmonary consolidation with obliteration of the costophrenic angle on the right. Direct sputum smear microscopy was positive for AFB, and the otorrhea culture for mycobacteria was positive for Koch's bacillus.

The patient was diagnosed with pulmonary tuberculosis and tuberculous otomastoiditis. A therapeutic regimen for tuberculosis was started with rifampicin, isoniazid, pyrazinamide, and ethambutol, as recommended by the Manual of Recommendations and Control of Tuberculosis in Brazil [6]. The intensive phase comprised rifampicin 450 mg/day, isoniazid 225 mg/day, pyrazinamide 1200 mg/day, and ethambutol 825 mg/day in the first two months. Subsequently, the patient received rifampicin 450 mg/day and isoniazid 225 mg/day for four months in the maintenance phase.

This patient did not require any surgical procedure. He showed a good clinical response to anti-tuberculosis (anti-TB) treatment. There was remission of otorrhea and granulation tissue, and the tympanic membrane regenerated spontaneously. After five years of follow-up, the EAC was widely patent, the tympanic membrane was opaque but intact, and the patient was clinically free of recurrence.

## Discussion

Tuberculous otitis media is an infection of the middle ear and mastoid cells caused by *Mycobacterium tuberculosis*. Non-pulmonary forms are responsible for 15-30% of tuberculosis cases, while tuberculous otitis media accounts for 0.3-3% of cases in the head and neck region [1]. Its spread can occur through hematogenous dissemination from distant places, entry of unwanted nasopharyngeal secretions/pathogens into the middle ear (by aspiration, insufflation, or reflux) by the Eustachian tube or direct implantation through the EAC and perforation of the tympanic membrane [2].

Patients initially present with painless, watery otorrhea that becomes profuse, thick, and purulent, resistant to conventional therapy, as in the cases presented. Hearing loss is frequent, which can occur early and from a severe to profound degree, disproportionate to otoscopic findings. Additionally, lymph node enlargement, cutaneous fistulas, and ipsilateral peripheral facial paralysis may occur [7]. Otoscopy may reveal multiple perforations that coalesce into a single large perforation or exuberant granulation tissue/inflammatory polyp occupying the EAC [3,8]. The two cases reported did not respond to the usual treatments with ear drops and developed irreversible severe/profound hearing loss. On otoscopy, the first case presented a double perforation with secretion and the presence of edema in the EAC and mucosal hyperplasia in the middle ear. The second had a polyp occupying the EAC and retroauricular lymph node enlargement. Neither case developed facial paralysis, abscess, or cutaneous fistulas.

Mastoid HRCT is the imaging test of choice for evaluating the temporal bone in patients with tuberculous otitis media. Magnetic resonance imaging of the temporal bone aids in the evaluation of complications. Imaging exams detect the presence of inflammatory tissue in the EAC, middle ear, and mastoid, the presence of osteitis with necrosis of the ossicles and areas of the temporal bone, and the presence of bone sequestration [2]. The attenuation of soft tissues throughout the middle ear cavity, the preservation of mastoid air cells without sclerotic changes, and the extension of the soft tissues into the EAC or thickening of the bony EAC mucosa were more associated with tuberculous otitis media than with suppurative chronic otitis media or cholesteatoma [7,9]. The cases presented had erosion of the cortical bone of the mastoid, posterior fossa, petrous apex, and inner ear, which could indicate a more advanced stage of the disease.

The diagnosis is made by identifying *Mycobacterium tuberculosis*. Bacilloscopy is a rapid and affordable method and is widely used. However, the concentration of bacilli in extrapulmonary forms is low, reducing the sensitivity of the test [2]. A culture of middle ear secretion or inflammatory tissue from the middle ear is considered the gold standard method. However, its positivity takes time. Molecular tests for DNA identification (e.g., RMT-TB) have been increasingly recommended. Although not very accessible, they are quick methods capable of detecting strains resistant to rifampicin [10]. In the case presented, these tests were performed and confirmed the diagnosis after positivity for *Mycobacterium tuberculosis*. Chest radiography/tomography are also tests that should be requested due to the frequent concomitant between pulmonary tuberculosis and chronic tuberculous otitis media [1,8,10].

The treatment is prolonged anti-TB chemotherapy with rifampicin, isoniazid, pyrazinamide, and ethambutol in the intensive phase and rifampicin and isoniazid for four months in the maintenance phase [6]. Surgical intervention is recommended when it is necessary to collect material for diagnosis, aeration of the mastoid, removal of the inflammatory process/bone sequestration, and in cases where complications are evident, such as the formation of abscesses, peripheral facial paralysis, infectious/serous labyrinthitis, pre- or post-auricular cutaneous fistula, involvement of the central nervous system, or associated cholesteatoma [5,10]. In the first case, the patient underwent canal wall-up tympanomastoidectomy and followed the recommended anti-TB treatment after surgery, presenting a good recovery. Conversely, the second patient did not require a surgical procedure and evolved with remission of otorrhea and granulation tissue only with anti-TB treatment.

## Conclusions

Tuberculous otitis media is a rare disease that poses diagnostic challenges due to insufficient clinical suspicion. It typically presents with painless otorrhea refractory to conventional antibiotic therapy. It is common to find profound hearing loss incompatible with otoscopy and erosion with bone sequestration in an imaging scan. The diagnosis is made with the detection of *Mycobacterium tuberculosis* in secretions from the middle ear or mastoid tissue. Treatment is carried out with anti-TB chemotherapy and possible surgical intervention. Knowledge about this pathology is of fundamental importance for early diagnosis and treatment before complications and sequelae appear.
